# Multilamellar Liposomes as a Model for Biological Membranes: Saturation Recovery EPR Spin-Labeling Studies

**DOI:** 10.3390/membranes12070657

**Published:** 2022-06-26

**Authors:** Witold Karol Subczynski, Marija Raguz, Justyna Widomska

**Affiliations:** 1 Department of Biophysics, Medical College of Wisconsin, Milwaukee, WI 53226, USA; 2 Department of Medical Physics and Biophysics, University of Split School of Medicine, 21000 Split, Croatia; 3 Department of Biophysics, Medical University of Lublin, 20 093 Lublin, Poland; justyna.widomska@umlub.pl

**Keywords:** multilamellar liposomes, saturation recovery EPR, membrane structure, membrane dynamics, membrane domains

## Abstract

EPR spin labeling has been used extensively to study lipids in model membranes to understand their structures and dynamics in biological membranes. The lipid multilamellar liposomes, which are the most commonly used biological membrane model, were prepared using film deposition methods and investigated with the continuous wave EPR technique (*T*_2_-sensitive spin-labeling methods). These investigations provided knowledge about the orientation of lipids, their rotational and lateral diffusion, and their rate of flip-flop between bilayer leaflets, as well as profiles of membrane hydrophobicity, and are reviewed in many papers and book chapters. In the early 1980s, the saturation recovery EPR technique was introduced to membrane studies. Numerous *T*_1_-sensitive spin-label methods were developed to obtain detailed information about the three-dimensional dynamic membrane structure. *T*_1_-sensitive methods are advantageous over *T*_2_-sensitive methods because the *T*_1_ of spin labels (1–10 μs) is 10 to 1000 times longer than the *T*_2_, which allows for studies of membrane dynamics in a longer time–space scale. These investigations used multilamellar liposomes also prepared using the rapid solvent exchange method. Here, we review works in which saturation recovery EPR spin-labeling methods were applied to investigate the properties of multilamellar lipid liposomes, and we discuss their relationships to the properties of lipids in biological membranes.

## 1. Short Overview of Previous Applications of EPR Spin Labeling in Studies of the Properties of Multilamellar Liposomes

**Figure 1:**
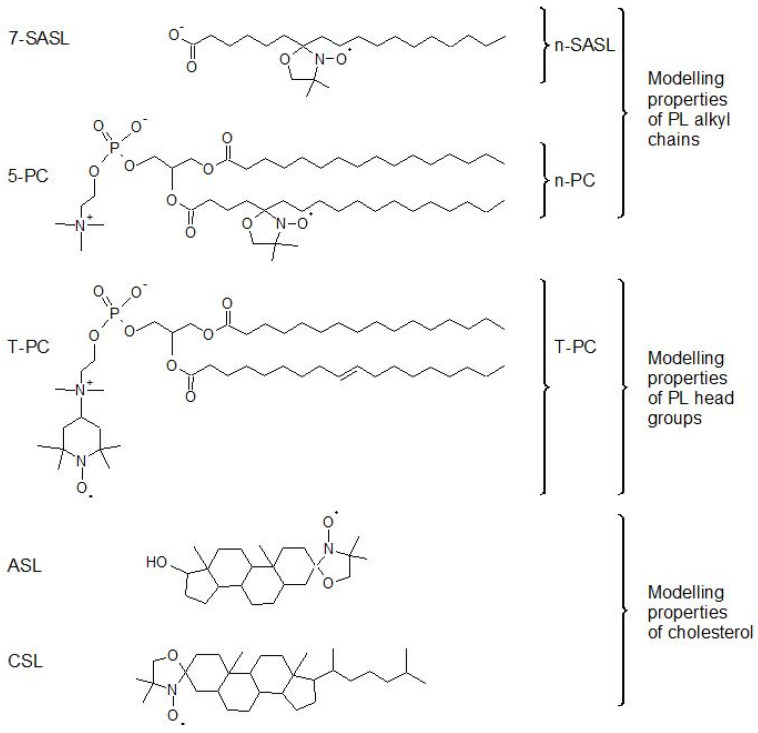
Chemical structures of three classes of lipid spin labels used in membrane studies. Stearic acid spin labels (*n*-SASL—n-doxylstearic acid spin label); PL spin labels (n-PC—1-palmitoyl-2-(n-doxylstearoyl)phosphatidylcholine and T-PC—tempocholine-1-palmitoyl-2-oleoylphosphatidic acid ester); and Chol-analog spin labels (ASL—androstane spin label and CSL—cholestane spin label).

The major nitroxide, free radical, lipid spin labels, which were later used in membrane studies, were first synthetized between 1967 and 1971. Primarily, three classes of these spin labels were used: spin-labeled stearic acids with the nitroxide fragment attached to different carbon atoms of the acyl chain (n-SASLs, synthesis described in [1,2,3]); spin-labeled phosphatidylcholines with the nitroxide fragment attached at selected positions on the fatty acid chain (n-PCs, synthesis described in [1]); or the polar head group (T-PC, synthesis described in [4]), and spin-labeled cholesterol (Chol) analogs (ASL, synthesis described in [5], and CSL, synthesis described in [6]) (see Figure 1 for their structures). The nitroxide free radicals, first synthetized by Hoffman (1961) [7] and by Rozantsev and Neiman (1964) [8] and used as monitoring groups in lipid spin labels, are extremely stable in model membranes. In addition, the basic approaches to analyze the electron paramagnetic resonance (EPR) spectra of these spin labels are rather simple, and the same for all of the spin labels [9,10,11]. The lipid spin labels are easily introduced to model membranes, while the lipids are mixed for the preparation of multilamellar liposomes. All of these broadly expanded the applications of EPR spin labeling for membrane studies.

As indicated in the abstract, the conventional continuous wave EPR techniques using phospholipid (PL) and Chol analog spin labels provide basic insights about the organization and dynamics of lipids in lipid bilayer membranes. In this review, we indicate the first major publications describing the dynamic properties of the bilayers, such as the acyl chain flexibility gradient and rotational diffusion [1,12,13], lipid lateral diffusion [2,14], and PL flip-flop between the leaflets of the bilayer [4,15]; where the phase transition between gel and fluid phase membranes was investigated [16,17,18]; where the order and orientation of lipids in the bilayer were investigated [10,19,20]; and where the shape of the transmembrane hydrophobic barrier was established with the spin-labeling approach [21,22]. These investigations are reviewed in many papers [23,24,25] and book chapters [26,27,28,29].

## 2. Background for the Application of SR EPR for Membrane Studies

The EPR techniques used to determine the structure and dynamics of model and biological membranes are continuously being developed to better understand membrane functions. In the early 1980s, the saturation recovery (SR) EPR technique was introduced for membrane studies. Numerous *T*_1_-sensitive spin-label methods were developed that greatly contributed to our understanding of the lateral organization of lipids in lipid bilayers, and to providing detailed information about their dynamics as a function of depth [30,31,32,33,34,35,36]. All of this information can be obtained at the physiological conditions for membranes (multilamellar liposomes) prepared from vastly diverse lipids, also resembling those forming the lipid bilayer portion of biological membranes. Here, we present a short theoretical background to show the applications of the SR EPR spin-labeling approaches to investigate model membranes.

**Figure 2:**
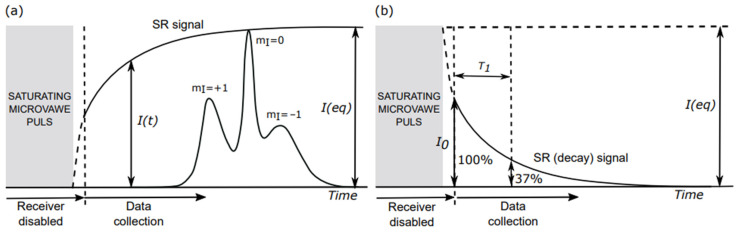
(**a**) The schematic illustration of the SR EPR experiment. The saturating high microwave power pump pulse (located at the field position of the central line (m_I_ = 0) of the spin-label absorption spectrum) of the electron spin system. If pump power is high enough, the amplitude of the signal decreases to zero (this is the case described in the Figure). The recovery signal to thermodynamic equilibrium is recorded at the same field position with a weak observing microwave power. The time of the “recovery disabled” is needed to protect the detector. The recovery to the equilibrium state is exponential, and is described by the equation, *I*(*t*) = *I*(eq) − *I*(eq)exp(−*t*/*T*_1_). *I*(*t*) and *I*(eq) are the amplitudes of the absorption spectrum at time *t* and at equilibrium, respectively. As indicated, the saturating pulse completely saturates the signal; (**b**) Usually, the SR signal is recorded and analyzed as a “decay” curve, which describes changes in the recorded EPR spectrum amplitude through the equation, SR signal = [*I*(eq) − *I*_0_]exp(−*t*/*T*_1_). Here, *I*_0_ is the amplitude of the signal at the beginning of the recording.

The SR EPR technique to measure the electron spin–lattice relaxation times (*T*_1_s) of paramagnetic samples (including nitroxide spin labels) was developed by Hyde in the 1970s [37,38]. With this technique, the recovery of the EPR signal to the thermodynamic equilibrium is recorded at a weak observing microwave power, not disturbing the EPR signal, and after the termination of the high saturating pump microwave power pulse (Figure 2a). The thermodynamic equilibrium is restored by the spin–lattice relaxation describing the interaction of the spin system with the “lattice” (close environment of spins). This recovery time is characterized by the spin–lattice relaxation time, *T*_1_. For convenience, the SR signal is recorded and analyzed as a “decay” curve (Figure 2b), which describes the changes in the EPR spectrum amplitude, through the equation:

*I*(*t*) = *I*_0_exp(−*t*/*T*_1_) (1)

*I*(*t*) and *I*_0_ are the amplitudes of the SR signal at time *t* and at the beginning of the recording. *T*_1_ is the spin–lattice relaxation time. For *t* = *T*_1_ (*t*/*T*_1_ = 1), the signal amplitude changes *e* times (SR signal decreases to 37% of its initial value). If the membrane domains are present with significantly different fluidities, the observed SR signal can be fitted to a multi-exponential function:

*I*(*t*) = ∑_n_*I*_0n_exp(−*t*/*T*_1n_), (2)

*I*_0n_ is the amplitude of the SR signal from each domain at the beginning of the recording, and *T*_1n_ is the spin–lattice relaxation time in each domain (see Section 6 for more explanation). 

For spin-labeled multilamellar liposomes, the SR EPR signals at the X-band (microwave frequency 9.4 GHz) are received on the central line (m_I_ = 0; see Figure 2a) of the EPR spin-label spectrum. To obtain the correct *T*_1_ values, the samples must be thoroughly deoxygenated (see Section 3). In this approach, which is used to obtain *T*_1_ values for single- and multi-exponential SR signals, it is not necessary for the pump pulse to be a saturating pulse. This qualitative SR EPR approach allows discrimination of the membrane domains. The SR EPR spin-labeling approach can also provide quantitative information about the lipids in the membrane domains (giving data about the relative amounts of PLs and Chol in the discriminated domains). The necessary condition for the quantitative approach is that the pump pulse completely saturates all of the components of the SR signal (see [39] for details of this method).

The factors that affect the measured *T*_1_ (in connection with the investigated system) are the structure of the nitroxide fragment, **rate of motion of the nitroxide fragment (rotational diffusion), collisions with oxygen and other paramagnetic relaxation agents**, dipole–dipole interaction with other paramagnetic agents, and local concentration of the spin labels. The factors that affect the measured *T*_1_ (in connection with the SR EPR measurement) are the observing microwave power and **microwave frequency**. The text in bold indicates the factors discussed in this review.

## 3. Preparation of Multilamellar Liposomes and Handling Samples for EPR Measurements

The multilamellar liposomes form preferred models in membrane studies. Most often, they are prepared using the film deposition method from the dry film of lipids and spin-label mixtures formed on the bottom of the test tube after the evaporation of the solvent, the addition of the buffer, and vortexing (shaking) [33]. Their multilamellar structure allows for the tight packing of model membranes in the sample capillary used for EPR measurements (as compared with the packing of the unilamellar liposomes), which greatly increases the signal-to-noise ratio of EPR signals and allows the concentration of the lipid spin labels in the investigated samples to be decreased to the magnetically diluted condition. The multilamellar liposomes can be easily pelleted in Eppendorf test tubes and further pelleted in the TPX or Teflon capillaries used for EPR measurements [40]. That way, the concentration of lipids in the aqueous sample used for EPR measurements can be as great as ~20% (wt/wt). The amount of water in these concentrated membrane suspensions is still high enough not to disturb the structure of the lipid bilayer [41]. Additionally, in the multilamellar dispersion, most liposomes have a diameter large enough that the effect of the membrane curvature on the properties of the inner and outer bilayer leaflets can be neglected. 

The film deposition methods, especially when applied to form multilamellar liposomes containing high amounts of Chol, have some limitations. As it dries, the lipid mixture passes the solid-state intermediate conditions at which the solid-state demixing of Chol, in the form of Chol crystals, can occur [42]. The Chol molecules from these crystals do not participate in membrane formation after the addition of the buffer because of the very high kinetic barrier. The rapid solvent exchange (RSE) method was designed to prevent the lipids from demixing during liposome preparation, as happens during preparations using the film deposition method [42]. During the preparation of the multilamellar liposomes using the RSE method, the lipid solutions never pass through the solid state, ensuring compositional homogeneity throughout the membrane suspension. The apparatus and the method are described in detail in [43,44]. Briefly, the chloroform solutions of lipid mixtures with an appropriate lipid spin label are added into the vortexed buffer, and vortexed further under reduced pressure to rapidly remove the solvent. The multilamellar liposomes obtained using the RSE method can be pelleted and transferred to sample capillaries the same way as those prepared using the film deposition method. The absence of demixing artifacts in the multilamellar liposomes produced using the RSE method makes it possible to obtain reproducible and precise Chol saturation limits in the different membranes (Chol concentration at which the pure Chol bilayer domains (CBDs) start to form) and confirms that the formation of CBDs precedes the formation of Chol crystals in the lipid bilayer membranes [45,46]. As indicated in Section 6, the CBDs can be discriminated with ASL and CSL, using the SR EPR technique and molecular oxygen (O_2_) or NiEDDA as the probe molecules.

**Figure 3:**
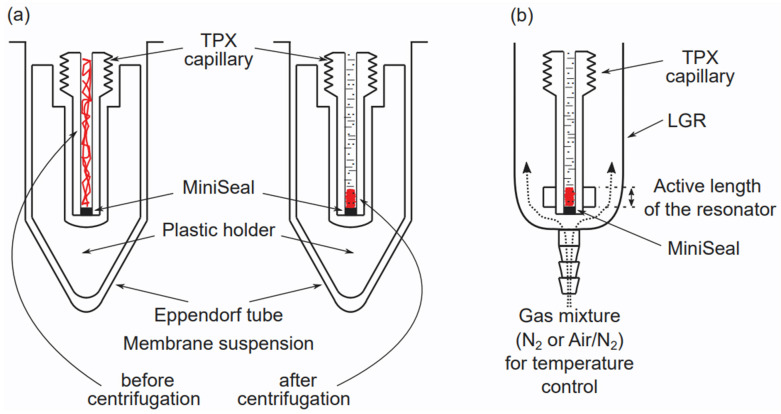
(**a**) This schematic drawing shows the basic strategy for handling samples of multilamellar liposomes made from a small amount of material. After the first concentrating (pelleting) of diluted samples to the volume of the TPX capillary, the suspension of multilamellar liposomes is further pelleted on the bottom of the TPX capillary to the lens of the active region of the loop-gap resonator (LGR). As shown in (**b**), the TPX capillary is then transferred from the special holder in the Eppendorf tube to the loop-gap resonator and equilibrated with the appropriate mixture of nitrogen and air; then, EPR measurements are performed with the possible maximal signal coming from that limited sample.

The basic handling of the multilamellar liposome samples is described in the first paragraph of this section. However, when the sample amount is very small, as for the multilamellar liposomes made from the total lipid extract of precious biological materials, some directions for handling can be useful. Introducing loop-gap resonators, especially for the SR EPR spectrometers, greatly reduces the active sample volume to a few microliters for X-band (microwave frequency 9.4 GHz) spectrometers, and to as little as 30 nL for Q-band (microwave frequency 34 GHz) and W-band (microwave frequency 94 GHz) spectrometers [47,48]. These approaches are described in [40]. Briefly, the preliminary work (before the EPR measurements) is performed for a convenient larger volume. After that, the samples are pelleted in Eppendorf tubes, and the pellet—still with a volume ten to a hundred times greater than that of the resonator’s active sample volume—is transferred to a TPX or Teflon capillary, where it is again pelleted on the bottom of the capillary to the lens of the active region of the resonator [48,49]. More details are presented in [40], and schematically in Figure 3a for the TPX capillary.

The basic strategies used to equilibrate the sample with the appropriate air/nitrogen mixture when the measurements are performed at X-band and W-band with TPX, quartz, and Teflon capillaries are explained in [50], and schematically in Figure 3b for the TPX capillary. 

## 4. Spin–Lattice Relaxation Rate as a Convenient Parameter of Membrane Fluidity

**Figure 4:**
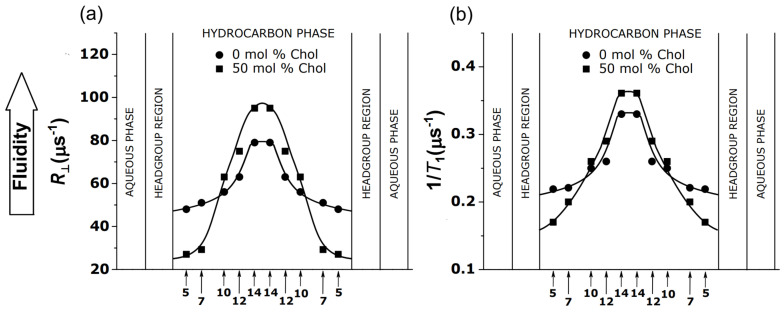
(**a**) Profiles of the rotational diffusion coefficient (*R*_⊥_) obtained with convenient continuous wave EPR technique and (**b**) spin–lattice relaxation rates (*T*_1_^−1^s) obtained with n-PC spin labels across DMPC membranes without and containing 50 mol% Chol using SR EPR technique. The nitroxide fragment position on the alkyl chain is indicated by “n.” Measurements were performed at 27 °C for thoroughly deoxygenated membranes. Values of *T*_1_^−1^ and *R*_⊥_ were taken from [57].

It is accepted that the spin–lattice relaxation rate (*T*_1_^−1^) of lipid spin labels mainly depends on the rate of the rotation of the nitroxide fragment attached to the parent lipid molecule [51,52,53]. Thus, *T*_1_^−1^ can be used as a convenient parameter to qualitatively describe the dynamics of the spin label (or, more specifically, the dynamics of the fragment of the parent lipid molecule to which the nitroxide fragment is rigidly attached). The measurements of *T*_1_^−1^ must be performed for deoxygenated samples. The detailed fluidity profiles of acyl chains can be obtained by measuring the *T*_1_^−1^ for n-PCs or n-SASLs with nitroxide fragments located at different depths in the membrane, using the SR EPR technique. These profiles qualitatively show the same detailed features as the profiles obtained from the simulation of the EPR spectra obtained with a convenient continuous wave EPR technique using the microscopic order and macroscopic disorder model [54,55,56], which provides spin-label rotational diffusion coefficients, an actual quantitative measure of the membrane fluidity [57,58]. Figure 4 illustrates the similarities between both of the profiles, obtained from the simulation of the EPR spectra and from measurements of *T*_1_^−1^, across the membranes of the multilamellar liposome dispersion of DMPC/Chol.

**Figure 5:**
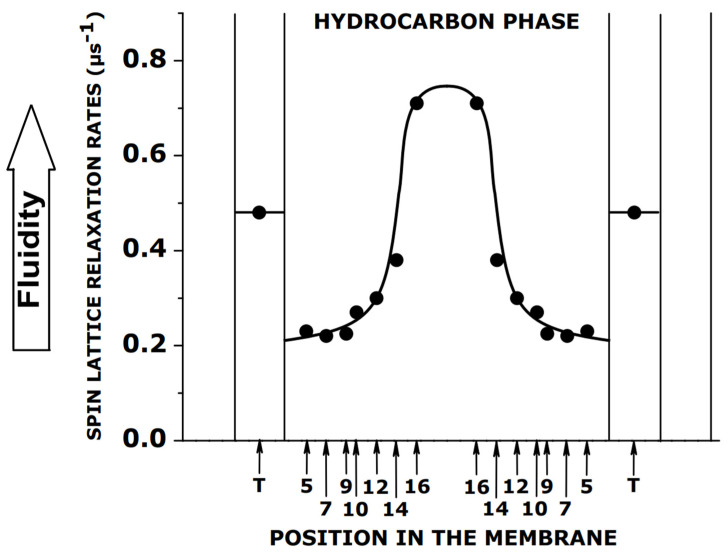
Profile of spin lattice relaxation rates (*T*_1_^−1^s) across membranes of multilamellar liposomes made of the lipid extracts pulled from cortical eye lenses of 61–70-year-old human donors. Measurements were performed at 37 °C for deoxygenated samples. Locations of spin labels for n-PCs are indicated by numbers and for T-PC by “T”. Based on the data obtained from the neutron diffraction studies on phosphatidylcholine model membranes [64,65] it is assumed that the location of the acyl chain carbon atom in the membrane changes linearly with the position on the acyl chain (the maximal error is about +/−1.5 Å). We also assumed that nitroxide moieties in n-PC are located at the same depth as appropriate carbon atoms of the 2-chain of PC membranes [66]. It can also be concluded that a nitroxide moiety stays at the position determined by neutron diffraction for most of the time. Adapted from [60], with permission of the Taylor and Francis Group.

The simulation of the EPR spectrum is not an easy process, as it depends on many parameters (including the principal values of the *g* tensors and hyperfine tensors) that should be determined for the particular environment in which the spin label is located. Because of that, the spin–lattice relaxation rate measurements, which are straightforward and easy to obtain, are very promising and already used to describe the profiles of membrane fluidity across model membranes of multilamellar liposomes made of simple lipids [57,58,59], as well as across the membranes of multilamellar liposomes made of the lipid extracts from biological membranes [50,60,61] (see Figure 5), and across the domains in complex biological membranes [62,63].

As indicated above, and as illustrated in Figure 4b and Figure 5, the *T*_1_^−1^ profiles provide excellent qualitative descriptions of the changes in membrane fluidity that occur after the additions of membrane modifiers. The results recently obtained in the Subczynski lab (not published) show a linear relationship between *T*_1_^−1^ and the rotational diffusion coefficients for the n-PC spin labels in the fluid-phase DMPC/Chol membranes of the multilamellar liposomes. The linearity is independent through the wide range of conditions, including the lipid environment, depth of membrane, local hydrophobicity, and anisotropy of rotational motion (independent of the order parameter). Thus, this linear dependence can be used as a calibration curve, allowing transformation qualitative profiles of *T*_1_^−1^ into quantitative profiles of spin-label rotational rates, which form a description of the membrane fluidity that is precise and easy to understand. Interestingly, a similar linear dependence between spin–lattice relaxation rates and rates of rotational motion was observed for a very different system, namely for various spin-labeled sites in the T4L protein (see Figure 2c in [67]).

## 5. Oxygen Transport Parameter as a Convenient Monitor of Membrane Fluidity

As shown in Section 4, the profiles of *T*_1_^−1^ can qualitatively describe membrane fluidity. As a result, the depth-dependent dynamics of the lipids forming the membrane can be acquired from the SR EPR measurements. All of these profiles are smooth and bell-shaped, which is the result of the cumulative effects of rotations that take place simultaneously at different positions along the chain. The movement of small molecules, such as O_2_, within the membrane is not affected by this cumulative effect. The molecular oxygen, a small and rather hydrophobic probe molecule, enters the vacant pockets transiently formed in the acyl chain matrix of membranes, moves with them, [68] and/or hops between the adjacent vacant pockets [69]. Because of that, the movement of O_2_ is sensitive to the dynamics of the acyl chains, and to the structural nonconformability of the neighboring lipids (see [35] for further explanation). 

The movement of O_2_ within the lipid bilayer membranes is monitored using the indirect SR EPR technique, in which the collision rate between paramagnetic oxygen molecules and the nitroxide fragment of lipid spin labels is measured. Kusumi et al. [33] developed an accurate and convenient way to evaluate this bimolecular collision rate using the SR EPR spin-labeling approach through the introduced oxygen transport parameter (OTP), defined as:

(3) $$\mathrm{OTP}={T_{1}}^{-1}(x,\;\mathrm{Air})-{T_{1}}^{-1}(x,\;N_{2})=AD(x)C(x)\;A=8\pi pr_{0}.$$

*T*_1_^−1^(x, Air) and *T*_1_^−1^(x, N_2_) are the spin–lattice relaxation rates of the spin labels for samples equilibrated with air and nitrogen, respectively. x is the depth of the location of the nitroxide in the membrane; *r*_0_ is the interaction distance between oxygen and the nitroxide (4.5 Å [70]); and *p* is the probability that an observable event occurs when a collision takes place. Thus, the OTP is the measure of the local oxygen diffusion-concentration product (*D*(x)*C*(x)) in the membrane around the nitroxide. This approach is quantitative because every collision of O_2_ with the nitroxide fragment contributes to the change in the nitroxide spin–lattice relaxation time [71]. By placing the nitroxide fragment of lipid spin labels at different depths in the membrane, the profiles of the OTPs (and, thus, the profiles of the oxygen diffusion-concentration products) across the membrane can be obtained.

**Figure 6:**
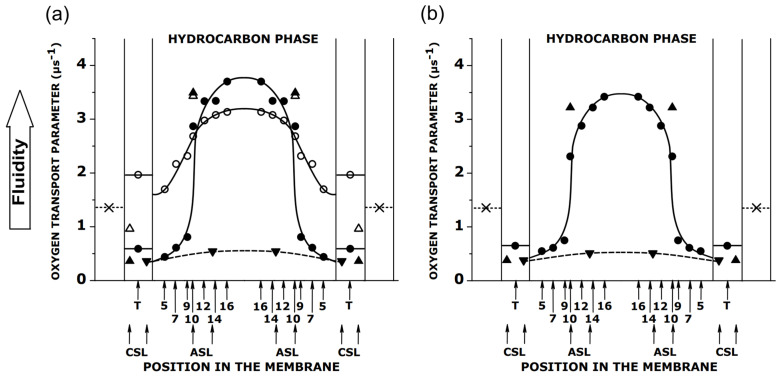
(**a**) Profiles of the oxygen diffusion-concentration products (OTPs) across membranes of multilamellar liposomes made of egg sphingomyelin in the absence (○,∆) and presence (●,▲,▼) of a saturating amount of Chol. Symbols (○,●) indicate profiles were obtained with n-PC, and symbols (∆,▲,▼) indicate data were obtained with ASL and CSL. Symbols (∆,▲) are for data from the PL bilayer, and symbols (▼) are for data from CBDs. It should be noted that the positions of the nitroxide moieties of ASL and CSL in the CBD are shifted toward the membrane center. A “sinking” of Chol molecules in the CBD was evaluated in [75] as about four C–C bonds deeper compared to Chol in the surrounding phospholipid bilayer; (**b**) Profiles of the OTPs across membranes of multilamellar liposomes made of the total lipid extracts from cortical fiber cell membranes of eye lenses from the group of 61–70-year-old human donors. Symbols (●,▲) indicate profiles were obtained with n-PC, ASL, and CSL in the PL bilayer, and symbols (▼) are for data obtained with ASL and CSL from CBDs. The symbol (x) indicates an OTP in water. All profiles were obtained at 37 °C. At ages ranging from 60 to 70 years, human lens fiber cell membranes contain ~70% sphingolipids [81]. Approximate locations of the nitroxide moieties of the spin labels are indicated by arrows (numbers for n-PC and “T” for T-PC). Data points for (**a**) are adapted from [82] with permission of the Elsevier Science & Technology Journals and for (**b**) are adapted from [60] with permission of the Taylor and Francis Group.

With the use of multilamellar liposomes, the OTP profiles were obtained across simple lipid bilayer membranes [34,35,72,73], as well as across the membranes of the multilamellar liposomes prepared from the total lipid extracts of biological membranes [60,61,74,75,76], and those formed from membranes reconstituted with integral membrane proteins [77]. A lack of the cumulative effect mentioned above allows for more detailed profiles of the membrane fluidity to be obtained, which have much greater spatial resolution than the profiles of the acyl chain motion. The OTP profiles show the membrane property changes with atomic resolution. As shown in Figure 6, the OTP profiles can indicate abrupt changes (i.e., changes that occur a few times) in membrane fluidity occurring on the distance of one C–C bond in the acyl chain. The abrupt change, which was observed for the membranes saturated with Chol, was recorded for the fluid phase membranes, indicating that the vertical fluctuation of the lipids must be diminished, and the lipids aligned (see [78,79] for further explanation). The molecular dynamics simulation also confirmed that the saturation with Chol increases the vertical order and smooths the phosphatidylcholine bilayer surface [80].

## 6. Discrimination by the OTP Method and Its Applications in Model Multilamellar Liposomes

OTP is a sensitive characteristic of membrane fluidity. Often, the fluidity of coexisting membrane domains cannot be differentiated in a straightforward manner by measuring the spin–lattice relaxation time (*T*_1_ values of spin labels in coexisting domains are too close to allow fitting the SR EPR signal to the double exponential). However, in the presence of O_2_, the collision of oxygen with nitroxide fragments of these spin labels can affect the *T*_1_ differently in both domains. Even small differences in the membrane lipid packing can affect the oxygen concentration and oxygen diffusion, which affect the *T*_1_s differently in both coexisting domains. In the method developed by Ashikawa et al. [77], the differences in *T*_1_s from the spin labels located in these two membrane domains and recorded in the presence of O_2_ allowed for differentiation between the domains. This method is named the discrimination by oxygen transport method. 

First, the discrimination by the oxygen transport method was used to discriminate the trapped lipid domain (slow oxygen transport domain) from the bulk plus boundary domain in the reconstituted membranes of bacteriorhodopsin and DMPC [77]. After that, it was used to discriminate raft domains [30] and CBDs [83]. Because the OTP profiles were obtained across these coexisting domains without their physical separation, few significant conclusions could be made. It is thought that the lipid–lipid interactions that form the liquid-ordered (*l*_o_) phase domains within the surrounding liquid-disordered (*l*_d_) bilayer may be responsible for the raft formation [84]. The OTP profiles across coexisting *l*_o_ and *l*_d_ phase domains [73] confirm that the properties of the *l*_o_ phase domain (modeling raft domain) lie between those for the *l*_d_ and the solid-ordered (*s*_o_) phases [85]. However, at the Chol saturation limit, the properties of the *l*_o_ phase to the depth of C9 are similar to those in the *s*_o_ phase. At depths greater than C9, they are similar to those in the *l*_d_ phase. The excess of Chol above the saturation limit of the *l*_o_ phase of the PL bilayer forms pure CBDs. These domains can be discriminated only with the Chol analog spin label, ASL, using O_2_ as a relaxation agent (see also Figure 6) [83]. The SR EPR signals of ASL from membranes contain these domains in the presence of O_2_; they can be fitted satisfactorily only by double exponential functions, giving two OTP values. As shown [60], the values of the OTPs in the bulk domain (surrounding CBDs) do not change with an increased Chol content. In the CBDs, these parameters are very close to those in the surrounding environment when the CBDs begin to form; however, as the Chol content increases, the parameters become increasingly separate from those in the surrounding domain. The most probable explanation for these observations is that the CBDs are small, just above the Chol saturation limit, and the Chol exchange between the coexisting domains strongly affects the properties of the small domain. When the size of an individual CBD increases, at a greater Chol content, the influence of the bulk domain decreases. The major conclusion is that, with increased Chol content, the amount of Chol in a CBD increases, as does the size of an individual CBD [60].

In the recent publication [86], the authors, using AFM with the measured tip of the radius of 32 nm, were not able to discriminate the CBDs in the supported lipid membranes made of Chol/POPC mixtures, up to a Chol/POPC mixing ratio of 3/1. They concluded that CBDs in these supported lipid membranes are small, smaller than the surface of the discriminating AFM tip (~3000 nm^2^).

## 7. Summary and Future Directions

The multilamellar liposomes are the model most often used to study the organization, dynamics, and properties of the lipid bilayer component of biological membranes. In addition, the EPR techniques, with the use of spin-labeling methods, have been applied extensively in these studies. The advancement of these techniques to the SR EPR technique [37,38] has allowed new information about the dynamics of lipids in membranes to be obtained. These investigations were possible because the spin-labeled multilamellar liposomes can be densely (tightly) packed in sample capillaries, allowing a good signal-to-noise ratio in the SR EPR measurements. In this review, we focus on recent developments that combine the RSE method of multilamellar liposomes’ preparation, SR EPR spin-labeling methods, and spin-label oximetry to study the three-dimensional dynamic structure of lipid bilayers to better understand its function in biological membranes. This combination forms a unique approach that allows an extension of the established phase diagram for Chol/DMPC mixtures [45] to the one-phase region where the CBDs are detected (the structured liquid-ordered phase) and the two-phase region where the structured liquid-ordered phase of DMPC coexists with Chol crystals. The approach developed to discriminate membrane domains in multilamellar liposomes (i.e., discrimination by the OTP method) has been used successfully to discriminate domains in complex biological membranes [62,63,87,88], and was further developed to quantify lipids in these domains [39]. 

The routine analysis of SR EPR signals from spin-labeled membranes using multi-exponential functions gives one, two, or—in some cases—three distinct spin–lattice relaxation rates for lipid spin labels, indicating that a membrane has one, two, or three distinct domains with distinct fluidities [33,73,83,88]. We think that the new way of analyzing the SR EPR signals of complex membranous systems using the stretched exponential function will allow more rigorous analysis of spin–lattice relaxation processes of lipid spin labels in membranes and parametrize the heterogeneity of membrane fluidity. This approach was first developed to analyze spin-label relaxation processes in complex biological membranes when one relaxation process (spin–lattice relaxations, due to the rotational diffusion of the spin labels) contributes to an exponential-like SR signal [89]. Recently, the second relaxation process, which contributes to the decay of SR signals, was included into the analysis, namely the Heisenberg exchange that occurs during collisions between the spin labels and O_2_ [90]. The obtained fitting parameters allow evaluation of not only the distribution (heterogeneity) of the rotational diffusions of spin labels in investigated membranes, but also the distribution (heterogeneity) of oxygen diffusion concentration products within the membrane. This approach adds new insight into membrane fluidity descriptions. Although this approach was developed to analyze the SR signals from complex biological membranes, it was also used to analyze SR signals from multilamellar liposomes made of total lipid extracts from these biological membranes, giving new information about the heterogeneity of their organization and their dynamics [90,91].

SR EPR spin labeling, as presently developed, forms an exceptional tool to study the organization (formation of membrane domains) and dynamics (profiles of membrane fluidity) of model and biological membranes. Previously, these studies mainly focused on the qualitative description of membrane characteristics. The new achievements indicate that these characteristics can be described in more quantitative ways. Thus, the principal applications of SR EPR spin-labeling methods in investigations of model and biological membranes can be broadened to quantitative measurements of membrane properties. 

## Data Availability

Not applicable.

## References

[1] Hubbell W.L., McConnell H.M. (1971). Molecular motion in spin-labeled phospholipids and membranes. J. Am. Chem. Soc..

[2] Devaux P., McConnell H.M. (1972). Lateral diffusion in spin-labeled phosphatidylcholine multilayers. J. Am. Chem. Soc..

[3] Waggoner A.S., Kingzett T.J., Rottschaefer S., Griffith O.H., Keith A.D. (1969). A spin-labeled lipid for probing biological membranes. Chem. Phys. Lipids.

[4] Kornberg R.D., McConnell H.M. (1971). Inside-outside transitions of phospholipids in vesicle membranes. Biochemistry.

[5] Hubbell W.L., McConnell H.M. (1969). Motion of steroid spin labels in membranes. Proc. Natl. Acad. Sci. USA.

[6] Keana J.F., Keana S.B., Beetham D. (1967). A new versatile ketone spin label. J. Am. Chem. Soc..

[7] Hoffmann A.K., Henderson A.T. (1961). A new stable free radical: Di-t-Butyl nitroxide. J. Am. Chem. Soc..

[8] Rozantsev E.G., Neiman M.B. (1964). Organic radical reactions involving no free valence. Tetrahedron.

[9] Schindler H., Seelig J. (1973). EPR spectra of spin labels in lipid bilayers. J. Am. Chem. Soc..

[10] Gaffney B.J., Berliner L.J. (1976). Practical considerations for the calculation of order parameter for fatty acid or phospholipid spin labels in membranes, Appendix IV. Spin Labeling. Theory and Applications.

[11] Berliner L.J. (1978). Spin labeling in enzymology: Spin-labeled enzymes and proteins. Methods Enzymol..

[12] McConnell H.M., McFarland B.G. (1972). The Flexibility Gradient in Biological Membranes. Ann. N. Y. Acad. Sci..

[13] Jost P., Libertini L.J., Hebert V.C., Griffith O.H. (1971). Lipid spin labels in lecithin multilayers. A study of motion along fatty acid chains. J. Mol. Biol..

[14] Kornberg R.D., McConnell H.M. (1971). Lateral diffusion of phospholipids in a vesicle membrane. Proc. Natl. Acad. Sci. USA.

[15] McNamee M.G., McConnell H.M. (1973). Transmembrane potentials and phospholipid flip-flop in excitable membrane vesicles. Biochemistry.

[16] Sackmann E., Trauble H. (1972). Studies of the crystalline-liquid crystalline phase transition of lipid model membranes. II. Analysis of electron spin resonance spectra of steroid labels incorporated into lipid membranes. J. Am. Chem. Soc..

[17] Sackmann E., Trauble H. (1972). Studies of the crystalline-liquid crystalline phase transition of lipid model membranes. I. Use of spin labels and optical probes as indicators of the phase transition. J. Am. Chem. Soc..

[18] Trauble H., Sackmann E. (1972). Studies of the crystalline-liquid crystalline phase transition of lipid model membranes. 3. Structure of a steroid-lecithin system below and above the lipid-phase transition. J. Am. Chem. Soc..

[19] Hubbell W.L., McConnell H.M. (1969). Orientation and motion of amphiphilic spin labels in membranes. Proc. Natl. Acad. Sci. USA.

[20] Libertini L.J., Waggoner A.S., Jost P.C., Griffith O.H. (1969). Orientation of lipid spin labels in lecithin multilayers. Proc. Natl. Acad. Sci. USA.

[21] Griffith O.H., Dehlinger P.J., Van S.P. (1974). Shape of the hydrophobic barrier of phospholipid bilayers (evidence for water penetration in biological membranes). J. Membr. Biol..

[22] Subczynski W.K., Wisniewska A., Yin J.J., Hyde J.S., Kusumi A. (1994). Hydrophobic barriers of lipid bilayer membranes formed by reduction of water penetration by alkyl chain unsaturation and cholesterol. Biochemistry.

[23] McConnell H.M., McFarland B.G. (1970). Physics and chemistry of spin labels. Q. Rev. Biophys..

[24] Keith A.D., Sharnoff M., Cohn G.E. (1973). A summary and evaluation of spin labels used as probes for biological membrane structure. Biochim. Biophys. Acta.

[25] Schreier S., Polnaszek C.F., Smith I.C. (1978). Spin labels in membranes. Problems in practice. Biochim. Biophys. Acta.

[26] Gaffney B.J., McNamee C.M. (1974). Spin-label measurements in membranes. With appendix: A use of computers in EPR spectroscopy. Methods Enzymol..

[27] Jost P., Waggoner A.S., Griffith O.H., Rothfield L. (1971). Spin labeling and membrane structure. Structure and Function of Biological Membranes.

[28] Melhorn R.J., Keith A.D., Fox C.F., Keith A.D. (1972). Spin-labeling biological membranes. Membrane Molecular Biology.

[29] Marsh D., Grell E. (1981). Electron Spin resonance: Spin labels. Membrane Spectroscopy.

[30] Kawasaki K., Yin J.J., Subczynski W.K., Hyde J.S., Kusumi A. (2001). Pulse EPR detection of lipid exchange between protein-rich raft and bulk domains in the membrane: Methodology development and its application to studies of influenza viral membrane. Biophys. J..

[31] Popp C.A., Hyde J.S. (1982). Electron-electron double resonance and saturation-recovery studies of nitroxide electron and nuclear spin-lattice relaxation times and Heisenberg exchange rates: Lateral diffusion in dimyristoyl phosphatidylcholine. Proc. Natl. Acad. Sci. USA.

[32] Yin J.J., Pasenkiewicz-Gierula M., Hyde J.S. (1987). Lateral diffusion of lipids in membranes by pulse saturation recovery electron spin resonance. Proc. Natl. Acad. Sci. USA.

[33] Kusumi A., Subczynski W.K., Hyde J.S. (1982). Oxygen transport parameter in membranes as deduced by saturation recovery measurements of spin-lattice relaxation times of spin labels. Proc. Natl. Acad. Sci. USA.

[34] Subczynski W.K., Hyde J.S., Kusumi A. (1989). Oxygen permeability of phosphatidylcholine--cholesterol membranes. Proc. Natl. Acad. Sci. USA.

[35] Subczynski W.K., Hyde J.S., Kusumi A. (1991). Effect of alkyl chain unsaturation and cholesterol intercalation on oxygen transport in membranes: A pulse ESR spin labeling study. Biochemistry.

[36] Yin J.J., Hyde J.S. (1987). Spin-label saturation-recovery electron spin resonance measurements of oxygen transport in membranes. Z. Für Phys. Chem..

[37] Huisjen M., Hyde J.S. (1974). A pulsed EPR spectrometer. Rev. Sci. Instrum..

[38] Percival P.W., Hyde J.S. (1975). Pulsed EPR spectrometer, II. Rev. Sci. Instrum..

[39] Mainali L., Camenisch T.G., Hyde J.S., Subczynski W.K. (2017). Saturation recovery EPR spin-labeling method for quantification of lipids in biological membrane domains. Appl. Magn. Reson..

[40] Subczynski W.K., Felix C.C., Klug C.S., Hyde J.S. (2005). Concentration by centrifugation for gas exchange EPR oximetry measurements with loop-gap resonators. J. Magn. Reson..

[41] Gennis R.B., Cantor C.R. (1989). Biomembranes. Molecular Structure and Function.

[42] Huang J., Buboltz J.T., Feigenson G.W. (1999). Maximum solubility of cholesterol in phosphatidylcholine and phosphatidylethanolamine bilayers. Biochim. Biophys. Acta.

[43] Buboltz J.T., Feigenson G.W. (1999). A novel strategy for the preparation of liposomes: Rapid solvent exchange. Biochim. Biophys. Acta.

[44] Buboltz J.T. (2009). A more efficient device for preparing model-membrane liposomes by the rapid solvent exchange method. Rev. Sci. Instrum..

[45] Mainali L., Raguz M., Subczynski W.K. (2013). Formation of cholesterol bilayer domains precedes formation of cholesterol crystals in cholesterol/dimyristoylphosphatidylcholine membranes: EPR and DSC studies. J. Phys. Chem. B.

[46] Mainali L., Pasenkiewicz-Gierula M., Subczynski W.K. (2020). Formation of cholesterol Bilayer Domains Precedes Formation of Cholesterol Crystals in Membranes Made of the Major Phospholipids of Human Eye Lens Fiber Cell Plasma Membranes. Curr. Eye Res..

[47] Froncisz W., Oles T., Hayde J.S. (1986). Q-band loop–gap resonator. Rev. Sci. Instrum..

[48] Hyde J.S., Yin J.J., Subczynski W.K., Camenisch T.G., Ratke J.J., Froncisz W. (2004). Spin-label EPR T1 values using saturation recovery from 2 to 35 GHz. J. Phys. Chem..

[49] Froncisz W., Hyde J.S. (1982). The loop–gap resonator: A new micro-wave lumped circuit ESR sample structure. J. Magn. Reson..

[50] Mainali L., Sidabras J.W., Camenisch T.G., Ratke J.J., Raguz M., Hyde J.S., Subczynski W.K. (2014). Spin-label W-band EPR with seven-loop-six-gap resonator: Application to lens membranes derived from eyes of a single donor. Appl. Magn. Reson..

[51] Marsh D. (2018). Molecular order and T1-relaxation, cross-relaxation in nitroxide spin labels. J. Magn. Reson..

[52] Mailer C., Nielsen R.D., Robinson B.H. (2005). Explanation of spin-lattice relaxation rates of spin labels obtained with multifrequency saturation recovery EPR. J. Phys. Chem. A.

[53] Robinson B.H., Haas D.A., Mailer C. (1994). Molecular dynamics in liquids: Spin-lattice relaxation of nitroxide spin labels. Science.

[54] Schneider D.J., Freed J.H., Berliner L.J. (1989). Calculating slow motional magnetic resonance spectra: A user’s guide. Spin Labeling: Theory and Application.

[55] Meirovitch E., Freed J.H. (1984). Analysis of slow-motional electron spin resonance spectra in smectic phases in terms of molecular configuration, intermolecular interactions, and dynamics. J. Phys. Chem..

[56] Earle K.A., Budil D.E., Schlick S. (2006). Calculating slow-motion ESR spectra of spin-labeled polymers. Advanced ESR Methods in Polymer Research.

[57] Mainali L., Feix J.B., Hyde J.S., Subczynski W.K. (2011). Membrane fluidity profiles as deduced by saturation-recovery EPR measurements of spin-lattice relaxation times of spin labels. J. Magn. Reson..

[58] Mainali L., Hyde J.S., Subczynski W.K. (2013). Using spin-label W-band EPR to study membrane fluidity profiles in samples of small volume. J. Magn. Reson..

[59] Mainali L., Raguz M., Subczynski W.K. (2012). Phases and domains in sphingomyelin-cholesterol membranes: Structure and properties using EPR spin-labeling methods. Eur. Biophys. J..

[60] Mainali L., Raguz M., O’Brien W.J., Subczynski W.K. (2017). Changes in the Properties and Organization of Human Lens Lipid Membranes Occurring with Age. Curr. Eye Res..

[61] Mainali L., Raguz M., O’Brien W.J., Subczynski W.K. (2013). Properties of membranes derived from the total lipids extracted from the human lens cortex and nucleus. Biochim. Biophys. Acta.

[62] Raguz M., Mainali L., O’Brien W.J., Subczynski W.K. (2015). Lipid domains in intact fiber-cell plasma membranes isolated from cortical and nuclear regions of human eye lenses of donors from different age groups. Exp. Eye Res..

[63] Raguz M., Mainali L., O’Brien W.J., Subczynski W.K. (2014). Lipid-protein interactions in plasma membranes of fiber cells isolated from the human eye lens. Exp. Eye Res..

[64] Zaccai G., Buldt G., Seelig A., Seelig J. (1979). Neutron diffraction studies on phosphatidylcholine model membranes. II. Chain conformation and segmental disorder. J. Mol. Biol..

[65] Janiak M.J., Small D.M., Shipley G.G. (1976). Nature of the Thermal pretransition of synthetic phospholipids: Dimyristolyl- and dipalmitoyllecithin. Biochemistry.

[66] Widomska J., Raguz M., Subczynski W.K. (2007). Oxygen permeability of the lipid bilayer membrane made of calf lens lipids. Biochim. Biophys. Acta.

[67] Yang Z., Bridges M., Lerch M.T., Altenbach C., Hubbell W.L. (2015). Saturation Recovery EPR and Nitroxide Spin Labeling for Exploring Structure and Dynamics in Proteins. Methods Enzymol..

[68] Trauble H. (1971). The movement of molecules across lipid membranes: A molecular theory. J. Membr. Biol..

[69] Pace R.J., Chan S.I. (1982). Molecular motions in lipid bilayers. III. Lateral and transverse diffusion in bilayers. J. Chem. Phys..

[70] Windrem D.A., Plachy W.Z. (1980). The diffusion-solubility of oxygen in lipid bilayers. Biochim. Biophys. Acta.

[71] Hyde J.S., Subczynski W.K., Berliner L.J. (1990). Spin-Label Oximetry. Biological Magnetic Resonance. Vol. 8. Spin Labeling. Theory and Applications.

[72] Subczynski W.K., Lewis R.N., McElhaney R.N., Hodges R.S., Hyde J.S., Kusumi A. (1998). Molecular organization and dynamics of 1-palmitoyl-2-oleoylphosphatidylcholine bilayers containing a transmembrane alpha-helical peptide. Biochemistry.

[73] Subczynski W.K., Wisniewska A., Hyde J.S., Kusumi A. (2007). Three-dimensional dynamic structure of the liquid-ordered domain in lipid membranes as examined by pulse-EPR oxygen probing. Biophys. J..

[74] Widomska J., Raguz M., Dillon J., Gaillard E.R., Subczynski W.K. (2007). Physical properties of the lipid bilayer membrane made of calf lens lipids: EPR spin labeling studies. Biochim. Biophys. Acta.

[75] Raguz M., Widomska J., Dillon J., Gaillard E.R., Subczynski W.K. (2008). Characterization of lipid domains in reconstituted porcine lens membranes using EPR spin-labeling approaches. Biochim. Biophys. Acta.

[76] Mainali L., Raguz M., O’Brien W.J., Subczynski W.K. (2015). Properties of membranes derived from the total lipids extracted from clear and cataractous lenses of 61-70-year-old human donors. Eur. Biophys. J..

[77] Ashikawa I., Yin J.J., Subczynski W.K., Kouyama T., Hyde J.S., Kusumi A. (1994). Molecular organization and dynamics in bacteriorhodopsin-rich reconstituted membranes: Discrimination of lipid environments by the oxygen transport parameter using a pulse ESR spin-labeling technique. Biochemistry.

[78] Raguz M., Widomska J., Dillon J., Gaillard E.R., Subczynski W.K. (2009). Physical properties of the lipid bilayer membrane made of cortical and nuclear bovine lens lipids: EPR spin-labeling studies. Biochim. Biophys. Acta.

[79] Subczynski W.K., Raguz M., Widomska J. (2010). Studying lipid organization in biological membranes using liposomes and EPR spin labeling. Methods Mol. Biol..

[80] Plesnar E., Subczynski W.K., Pasenkiewicz-Gierula M. (2012). Saturation with cholesterol increases vertical order and smoothes the surface of the phosphatidylcholine bilayer: A molecular simulation study. Biochim. Biophys. Acta.

[81] Deeley J.M., Mitchell T.W., Wei X., Korth J., Nealon J.R., Blanksby S.J., Truscott R.J. (2008). Human lens lipids differ markedly from those of commonly used experimental animals. Biochim. Biophys. Acta.

[82] Mainali L., Raguz M., Subczynski W.K. (2011). Phase-separation and domain-formation in cholesterol-sphingomyelin mixture: Pulse-EPR oxygen probing. Biophys. J..

[83] Raguz M., Mainali L., Widomska J., Subczynski W.K. (2011). Using spin-label electron paramagnetic resonance (EPR) to discriminate and characterize the cholesterol bilayer domain. Chem. Phys. Lipids.

[84] Simons K., Vaz W.L. (2004). Model systems, lipid rafts, and cell membranes. Annu. Rev. Biophys. Biomol. Struct..

[85] Loura L.M., Fedorov A., Prieto M. (2001). Fluid-fluid membrane microheterogeneity: A fluorescence resonance energy transfer study. Biophys. J..

[86] Khadka N.K., Timsina R., Rowe E., O’Dell M., Mainali L. (2021). Mechanical properties of the high cholesterol-containing membrane: An AFM study. Biochim. Biophys. Acta Biomembr..

[87] Mainali L., Raguz M., O’Brien W.J., Subczynski W.K. (2012). Properties of fiber cell plasma membranes isolated from the cortex and nucleus of the porcine eye lens. Exp. Eye Res..

[88] Mainali L., O’Brien W.J., Subczynski W.K. (2019). Detection of cholesterol bilayer domains in intact biological membranes: Methodology development and its application to studies of eye lens fiber cell plasma membranes. Exp. Eye Res..

[89] Stein N., Mainali L., Hyde J.S., Subczynski W.K. (2019). Characterization of the distribution of spin-lattice relaxation rates of lipid spin labels in fiber cell plasma membranes of eye lenses with a stretched-exponential function. Appl. Magn. Reson..

[90] Stein N., Subczynski W.K. (2021). Oxygen Transport Parameter in Plasma Membrane of Eye Lens Fiber Cells by Saturation Recovery EPR. Appl. Magn. Reson..

[91] Stein N., Subczynski W.K. (2021). Differences in the properties of porcine cortical and nuclear fiber cell plasma membranes revealed by saturation recovery EPR spin labeling measurements. Exp. Eye Res..

